# Increased risk of developing cerebro-cardiovascular diseases in police officers: a nationwide retrospective cohort study

**DOI:** 10.1186/s40885-024-00277-6

**Published:** 2024-07-01

**Authors:** Juyeon Ko, Hyunji Park, Sungha Park, Dae-hee Kim, Jaelim Cho

**Affiliations:** 1 Department of Preventive Medicine, Yonsei University College of Medicine, Seoul, Republic of Korea; 2 Division of Cardiology, Severance Cardiovascular Hospital, Yonsei University College of Medicine, Seoul, Republic of Korea; 3 Division of Cardiology, Department of Internal Medicine, Asan Medical Center, University of Ulsan College of Medicine, Seoul, Republic of Korea; 4 Institute for Environmental Research, Yonsei University College of Medicine, Seoul, Republic of Korea; 5 Institute of Human Complexity and Systems Science, Yonsei University, Incheon, Republic of Korea

**Keywords:** Cerebrovascular diseases, Cohort, Police, Public officer, Hypertension

## Abstract

**Background:**

Police officers face an increased risk of developing cerebro-cardiovascular diseases (CVD). However, current literature lacks population-based cohort studies specifically focusing on this association. This study aimed to investigate the association between police officers and the risk of developing CVD compared with education officers, while accounting for socioeconomic and demographic factors.

**Methods:**

We used the Korean National Health Insurance Service data spanning from 2009 to 2020. In this population-based retrospective matched cohort study, we identified age, sex, and calendar years of job-enrollment–matched education officers for each police officer. This study evaluated the CVD occurrence, including acute myocardial infarction, ischemic stroke, and hemorrhagic stroke. Using multivariable Cox regression analysis, we determined the risk of developing CVD, expressed as a hazard ratio (HR) and 95% confidence interval (CI).

**Results:**

Among 104,134 police officers and 104,134 education officers, 4,391(42.2%) cases and 3,631(34.9%) cases of CVD occurred, respectively. The mean ± standard deviation age was 38.4 ± 9.4 years in police officers and 38.6 ± 9.5 years in education officers. The proportion of men was 84.8 % in both groups. Police officers were significantly associated with a higher risk of developing CVD compared with education officers, with an adjusted HR of 1.15 (95% CI, 1.09–1.22). In addition, police officers had significantly higher risks for acute myocardial infarction (adjusted HR, 1.16; 95% CI, 1.06–1.26) and ischemic stroke (adjusted HR, 1.17; 95% CI, 1.09–1.25).

**Conclusions:**

The findings of our study highlight a significant increase in the risk of developing CVD among police officers, particularly among those aged 45 years and older and those with uncontrolled blood pressure compared to their education officer counterparts. Future cohort studies are required to confirm this association.

**Supplementary Information:**

The online version contains supplementary material available at 10.1186/s40885-024-00277-6.

## Background

Police officers face various health issues that influence their overall well-being, including an elevated risk of developing cerebro-cardiovascular diseases (CVD) [1–4]. A population-based study in the Republic of Korea showed that police officers had a significantly higher risk of developing angina pectoris (1.5 times higher), cerebrovascular disease (1.4 times higher), and acute myocardial infarction (1.8 times higher) compared with other government workers [2]. Similarly, a retrospective study from the United States showed 52.6% of deaths attributed to CVD occurred among police officers [3]. By contrast, another study conducted in the United States, comparing police officers to the general US population, found no significant associations between job-related deaths due to CVD among police officers, particularly in relation to ischemic heart disease and cerebrovascular disease [1].

Numerous studies showing an increased risk of developing CVD among police officers [2–5]. However, these findings derived from studies where police officers were part of larger study groups, with CVD not being the primary outcome [2–5]. Thus, large-scale cohort studies specifically investigating the association between police officers and CVD risk as the primary outcome are lacking. Furthermore, previous investigations have often inadequately considered influential variables [2, 4, 6], such as socioeconomic status, and modifiable factors, such as body mass index (BMI), which may confound the relationship between police officers and CVD risk. Moreover, inconsistent results were reported, with one study demonstrating null association between police officers and job-related CVD deaths [1].

This study aimed to investigate the association between police officers and the risk of developing CVD while accounting for socioeconomic and demographic variations and compare this risk with that of education officers.

## Methods

### Ethics statement

This study was approved by the Institutional Review Board of Yonsei University Health System (No. 4-2022-0436). The present study acquired data from the National Health Insurance Service in the Republic of Korea. The analytic code will be made available from the corresponding authors on request. Since the data were de-identified, the requirement for written informed consent was waived.

### Data source

We conducted a cohort focused on public officers (including police and education officers) identified between 2006 and 2020 using data from the Korean National Health Insurance Service. The Korean National Health Insurance Service data cover outpatient and inpatient care, as well as national health examinations, in almost 100% of the Korean population. The outpatient and inpatient care data contained information on demographics, primary and secondary diagnoses (using the International Classification of Diseases, 10th Revision [ICD-10]), diagnosis date, and death date. The national health examination data included demographics (age, sex, and socioeconomic status), anthropometrics (height, weight, and blood pressure), laboratory results (fasting blood glucose and total cholesterol levels), and lifestyle behaviors (alcohol consumption, smoking status, and physical activity).

### Study cohort

**Fig. 1:**
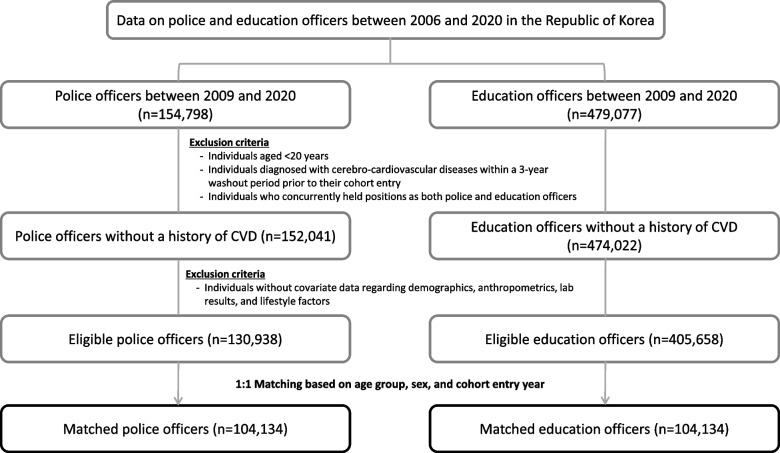
Selection process for the study participants. CVD, Cerebro-cardiovascular diseases

In this population-based retrospective matched cohort study, we identified 633,875 public officers (police and education officers) based on occupational information (e.g., identification of public officers and workplaces). A total of 154,798 police officers were identified during the study period from January 1, 2009, to December 31, 2020 (Fig. 1). To ensure that the police officers were newly diagnosed with CVD we applied a 3-year washout period before the cohort entry. The date of first identification as a police officer was set as the cohort entry date using January 1 and their calendar year of job enrollment. The exclusion criteria used in this study were as follows: individuals aged <20 years; diagnosed with CVD (defined as acute myocardial infarction [ICD-10, I21–I22], ischemic stroke [I63], and hemorrhagic stroke [I60–62]) within the 3-year washout period prior to the cohort entry; who concurrently held positions as both police and education officers; and without covariate data on demographics, anthropometrics, laboratory results, and lifestyle factors.

The included covariates were selected based on a specific priority scheme. Data on the year of cohort entry held the highest priority (first choice), followed by 1 year before (second choice), and 1 year after the cohort entry (third choice). Additionally, data from 1 year (fourth choice), 3 years (fifth choice), and 4 years (sixth choice) after the cohort entry were considered for inclusion in the analysis. Subsequently, 130,938 individuals were deemed eligible and included in the police officer group for analysis. A total of 479,077 education officers were identified from 2009 to 2020; the same exclusion criteria, and the 3-year washout period were applied. Of these, for each police officer, based on the age group, sex, and cohort entry year, one education officer was randomly selected for matching (208,268 in total; 104,134 per group). Age group was stratified into 5-year intervals, creating nine subgroups (e.g., age 20–24 years and 25–29 years) that were used to perform frequency matching of each education officer to each police officer.

### Follow-up

The endpoint was CVD development, including acute myocardial infarction, ischemic stroke, and hemorrhagic stroke. We defined CVD development as the first hospital admission for any of the above three diseases during the follow-up period between 2009 and 2020. Additionally, for each disease within the CVD category, the development was defined based on the first admission for that particular disease during the same follow-up period. Follow-up ended with the development of CVD, death, or the end of the study period (December 31, 2020), whichever occurred first.

### Other variables

This cohort study considered demographics (age and socioeconomic status), lifestyle behaviors (alcohol consumption and smoking status), BMI, total cholesterol level, and hypertension and diabetes status as main covariates.

The demographic information included age (as a continuous variable) and health insurance rate (as a proxy for socioeconomic status). Lifestyle behaviors included alcohol consumption (in frequency) and smoking status (never smoked, former smoker, or current smoker). BMI (kg/m^2^) was calculated by dividing weight (kg) by height (m^2^). Both BMI and total cholesterol level were treated as a continuous variable.

Hypertension status was categorized as diagnosed hypertension, undiagnosed hypertension, and no hypertension. Diagnosed hypertension is defined as having at least one relevant ICD-10 codes for hypertension (I10–I16) at primary and/or secondary positions in the past three years prior to cohort entry and/or systolic blood pressure exceeding 140 mmHg or diastolic blood pressure exceeding 90 mmHg in the year of cohort entry. Undiagnosed hypertension is defined as having systolic blood pressure exceeding 140 mmHg or diastolic blood pressure exceeding 90 mmHg without a prior hypertension diagnosis. Individuals who did not meet the criteria for either diagnosed or undiagnosed hypertension were classified as no hypertension.

Diabetes status was categorized as diagnosed diabetes, undiagnosed diabetes, and no diabetes. Diagnosed diabetes was defined as having at least one relevant ICD-10 code for diabetes (I20–I25, I48, I50, I60–I69) at primary and/or secondary positions in the past three years prior to cohort entry and/or fasting blood sugar levels ≥126 mg/dL in the year of cohort entry. Undiagnosed diabetes is defined as having fasting blood sugar levels ≥126 mg/dL without a prior hypertension diabetes. Individuals who did not meet the criteria for either diagnosed or undiagnosed diabetes were classified as no diabetes.

Additional covariates were defined as follows: a history of depressive disorders was defined as having at least one relevant ICD-10 code (F32.0–32.9, F33.0–33.3, F33.8, F33.9, F34.1) at primary and/or secondary positions in the past three years prior to cohort entry. Similarly, a history of posttraumatic stress disorder was defined as having at least one relevant ICD-10 codes (F43.10–43.12) at primary and/or secondary positions in the past three years prior to cohort entry.

Physical activity was assessed by a self-reported question: “How many days in the past week did you walk for at least 30 minutes in total, including any circumstances where you walked for at least 10 minutes at a time?” The response to this question was categorized as never, 1-2 times, 3-4 times, 5-6 times, daily, and unknown.

### Statistical analysis

Independent t-tests (for continuous variables), Wilcoxon signed rank tests (for total cholesterol only), and chi-square tests (for categorical variables) were conducted to examine differences in characteristics between police and education officers. Kaplan-Meier curves were plotted to compare the survival probability between police and education officers, and the significance of differences in survival probability was tested using a log-rank test. Crude and multivariable Cox regression analyses were performed to estimate the risk of developing CVD in police officers compared with education officers. The multivariable Cox regression model was adjusted for age, BMI, health insurance rate (quartile), total cholesterol level, alcohol consumption, smoking status, hypertension and diabetes status. Risk is expressed as a hazard ratio (HR) with a 95% confidence interval (CI). We also estimated the risk of developing each CVD component in police officers compared with education officers. Seven sensitivity analyses were performed. Two separate analyses were performed, each adjusting for physical activity and history of depressive disorders individually as additional covariates in the multivariable Cox regression model. Additionally, five subgroup analyses were conducted, stratified by age (20–44 years, ≥45 years), sex, BMI (<25 kg/m^2^, ≥25 kg/m^2^), hypertension status (normal, undiagnosed hypertension, diagnosed hypertension), and diabetes status (normal, undiagnosed diabetes, diagnosed diabetes). In these subgroup analyses, the subgroup variable was not adjusted for in the Cox regression model. A test of interaction in all subgroup analyses was performed according to the method described by Altman and Bland [7].

All analyses were performed using SAS ver. 9.4 (SAS Institute). Statistical significance was set at *P*<0.05.

## Results

### Baseline characteristics

A total of 208,268 police and education officers (104,134 individuals per group) were included. Among 104,134 police officers and 104,134 education officers, 4,391(42.2%) cases and 3,631(34.9%) cases of CVD occurred, respectively. The median follow-up period was 2,212 days (interquartile range, 1,091–3,362 days) for police officers and 2,318 days (interquartile range, 1,115–3,399 days) for education officers. The mean ± standard deviation age was 38.4 ± 9.4 years in police officers and 38.6 ± 9.5 years in education officers. The proportion of men was 84.8 % in both groups. There were no individuals diagnosed with posttraumatic stress disorder. The other characteristics are presented in Table 1 and Table S1.

**Table 1:** Baseline characteristics of the participants (*n* = 208,268)

Characteristic	Police officer (*n* = 104,134)	Education officer (*n* = 104,134)	*P*-value^a^
Age (yr)	38.4 ± 9.4	38.6 ± 9.5	0.001
Men	88,301 (84.8)	88,301 (84.8)	<0.999
Health insurance rate			<0.001
Quartile 1	25,080 (24.1)	19,187 (18.4)	
Quartile 2	27,294 (26.2)	32,870 (31.6)	
Quartile 3	25,920 (24.9)	18,949 (18.2)	
Quartile 4	25,062 (24.1)	30,987 (29.8)	
Missing	778 (0.8)	2,141 (2.1)	
Body mass index (kg/m^2^)	24.3 ± 2.9	23.7 ± 3.1	<0.001
Fasting blood glucose (mg/dL)	95.8 ± 22.0	94.1 ± 18.4	<0.001
Total cholesterol (mg/dL)	188.0 (167–212)	189.0 (168–213)	<0.001
Systolic blood pressure (mmHg)	121.8 ± 13.1	119.9 ±13.7	<0.001
Diastolic blood pressure (mmHg)	76.4 ± 9.5	75.1 ± 10.0	<0.001
Alcohol consumption			<0.001
Nondrinker	6,054 (5.8)	22,051 (21.2)	
1–2 times/wk	9,732 (9.4)	32,587 (31.3)	
3–4 times/wk	1,336 (1.3)	2,932 (2.8)	
Everyday	87,012 (83.6)	46,564 (44.7)	
Smoking status			<0.001
Never	45,370 (43.6)	60,486 (58.1)	
Ever-smoker	23,434 (22.5)	17,184 (16.5)	
Current smoker	35,330 (33.9)	26,464 (25.4)	
Physical activity (walking)			<0.001
None	5,614 (5.4)	18,939 (18.2)	
1-2 times/wk	7,267 (7.0)	23,889 (22.9)	
3-4 times/wk	3,165 (3.0)	10,318 (9.9)	
5-6 times/wk	475 (0.4)	1,921 (1.8)	
Daily	532 (0.5)	2,026 (1.9)	
Unknown	87,081 (83.6)	47,041 (45.2)	
Hypertension^b^	19,269 (18.5)	17,210 (16.6)	<0.001
Diabetes^c^	10,195 (9.8)	7,403 (7.1)	<0.001
History of depressive disorders^d^	2,794 (2.7)	3,067 (3.0)	<0.001

Data are presented as mean ± standard deviation, number (%), or median (interquartile range). Percentages may not total 100 due to rounding

^a^*P*-values were obtained using independent t-tests (for continuous, normally distributed variables), Wilcoxon rank-sum tests (for continuous non-normalized variables), and chi-square tests (for categorical variables) between police and education officers

^b^Hypertension status was defined as having at least one relevant ICD-10 codes for hypertension (I10–I16) at primary and/or secondary positions in the past three years prior to cohort entry and/or systolic blood pressure exceeding 140 mmHg or diastolic blood pressure exceeding 90 mmHg in the year of cohort entry

^c^Diabetes status was defined as having at least one relevant ICD-10 code for diabetes (I20–I25, I48, I50, I60–I69) at primary and/or secondary positions in the past three years prior to cohort entry and/or fasting blood sugar levels ≥126 mg/dL in the year of cohort entry

^d^History of depressive disorders was defined as having at least one relevant ICD-10 code (F32.0–32.9, F33.0–33.3, F33.8, F33.9, F34.1) at primary and/or secondary positions in the past three years prior to cohort entry

### Risk of developing CVD in police officers compared with education officers

The risk of developing CVD in police officers was significantly different from that in education officers. Police officers had the lowest survival probability compared with education officers (log-rank test, *P* < 0.001). Compared with education officers, police officers had significantly higher risks of developing CVD (adjusted HR, 1.15; 95% CI, 1.09–1.22), acute myocardial infarction (adjusted HR, 1.16; 95% CI, 1.06–1.26), and ischemic stroke (adjusted HR, 1.17; 95% CI, 1.09–1.25). However, the risk of developing hemorrhagic stroke in police officers was significantly different from that in education officers (Table 2).

**Table 2:** Risk of cerebro-cardiovascular diseases among police officers compared with education officers (*n* = 208,268)

Disease	No. of events (%)		Crude HR (95% CI)	Adjusted HR (95% CI)
	Police officer (*n* = 104,134)	Education officer (*n* = 104,134)		
Cerebro-cardiovascular disease	4,391 (4.2)	3,631 (3.5)	1.22 (1.16–1.27)	1.15 (1.09–1.22)
Acute myocardial infarction	1,542 (1.5)	1,257 (1.2)	1.23 (1.14–1.32)	1.16 (1.06–1.26)
Ischemic stroke	2,499 (2.4)	1,974 (1.9)	1.26 (1.18–1.33)	1.17 (1.09–1.25)
Hemorrhagic stroke	683 (0.7)	654 (0.6)	1.05 (0.94–1.16)	1.06 (0.93–1.19)

Adjusted HRs were estimated using multivariable Cox regression models, including adjustments for age, sex, body mass index, health insurance rate, total cholesterol, alcohol consumption, smoking status, hypertension and diabetes status. Total cholesterol variables were log-transformed

*HR* hazard ratio, *CI* confidence interval, *ICD-10* International Classification of Disease, 10th Revision

### Sensitivity analyses

In the sensitivity analysis including physical activity, police officers had a higher risk of developing CVD (HR, 1.25; 95% CI, 1.16–1.35) and ischemic stroke (HR, 1.34; 95% CI, 1.21–1.49) compared with education officers. The risk of developing acute myocardial infarction and hemorrhagic stroke were not statistically significant (data not shown).

In the sensitivity analysis including history of depressive disorders, police officers had a higher risk of developing CVD (HR, 1.15; 95% CI, 1.09–1.22), acute myocardial infarction (HR, 1.16; 95% CI, 1.06–1.27), and ischemic stroke (HR, 1.17; 95% CI, 1.09–1.26) compared with education officers. The risk of developing hemorrhagic stroke were not statistically significant (data not shown).

In the sensitivity analysis stratified by age, the risk of developing CVD (HR, 1.24; 95% CI, 1.16–1.33), acute myocardial infarction (HR, 1.14; 95% CI, 1.01–1.30), and ischemic stroke (HR, 1.29; 95% CI, 1.18–1.41) were higher in police officers with aged ≥45 years old compared with education officers. Age significantly modifies the association between police officers and their risk of developing CVD (police officers aged ≥45 years old vs. aged 20–44 years, P for interaction = 0.004) and ischemic stroke (police officers aged ≥45 years old vs. aged 20–44 years, P for interaction < 0.001). The other findings are presented in Table 3.

**Table 3:** Risk of cerebro-cardiovascular diseases stratified by age

Disease	HR (95% CI)		*P* for interaction^a^
	20–44 yr	≥45 yr	
Cerebro-cardiovascular disease	1.06 (0.97–1.15)	1.24 (1.16–1.33)	0.004
Acute myocardial infarction	1.22 (1.07–1.39)	1.14 (1.01–1.30)	0.477
Ischemic stroke	0.94 (0.83–1.07)	1.29 (1.18–1.41)	<0.001
Hemorrhagic stroke	1.02 (0.85–1.22)	1.10 (0.91–1.32)	0.557

Hazard ratios were estimated using multivariable Cox regression models, including adjustments for age, sex, body mass index, health insurance rate, total cholesterol, alcohol consumption, smoking status, hypertension and diabetes status. Total cholesterol variables were log-transformed

*HR* hazard ratio, *CI* confidence interval, *ICD-10* International Classification of Disease, 10th Revision

^a^Significant differences in the adjusted hazard ratios between the age groups, determined using the Bland-Altman method

In the sensitivity analysis stratified by hypertension status, the risk of developing CVD were higher in police officers with undiagnosed hypertension (HR, 1.35; 95% CI, 1.16–1.58), diagnosed hypertension (HR, 1.19; 95% CI, 1.07–1.32), and without hypertension (HR, 1.11; 95% CI, 1.03–1.18) compared with education officers. Hypertension status significantly modifies the association between police officers and their risk of developing CVD (police officers with undiagnosed hypertension vs. no hypertension, *P* for interaction = 0.016) and acute myocardial infarction (police officers with undiagnosed hypertension vs. no hypertension, *P* for interaction = 0.015). The other findings are presented in Table 4.

**Table 4:** Risk of cerebro-cardiovascular diseases stratified by hypertension status

Disease	HR (95% CI)			*P* for interaction^a^	*P* for interaction^b^
	No hypertension	Undiagnosed hypertension	Diagnosed hypertension		
Cerebro-cardiovascular disease	1.11 (1.03–1.18)	1.35 (1.16–1.58)	1.19 (1.07–1.32)	0.016	0.266
Acute myocardial infarction	1.13 (1.02–1.27)	1.62 (1.24–2.10)	1.04 (0.87–1.25)	0.015	0.441
Ischemic stroke	1.09 (0.99–1.20)	1.16 (0.95–1.42)	1.34 (1.17–1.54)	0.588	0.012
Hemorrhagic stroke	1.00 (0.85–1.16)	1.25 (0.88–1.76)	1.15 (0.87–1.51)	0.247	0.384

Hazard ratios were estimated using multivariable Cox regression models, including adjustments for age, sex, body mass index, health insurance rate, total cholesterol, alcohol consumption, smoking status, and diabetes status. Total cholesterol variables were log-transformed

Hypertension status was categorized as diagnosed hypertension, undiagnosed hypertension, and no hypertension. Diagnosed hypertension is defined as having at least one relevant ICD-10 codes for hypertension (I10–I16) at primary and/or secondary positions in the past three years prior to cohort entry and/or systolic blood pressure exceeding 140 mmHg or diastolic blood pressure exceeding 90 mmHg in the year of cohort entry. Undiagnosed hypertension is defined as having systolic blood pressure exceeding 140 mmHg or diastolic blood pressure exceeding 90 mmHg without a prior hypertension diagnosis. Individuals who did not meet the criteria for either diagnosed or undiagnosed hypertension were classified as no hypertension

*HR* hazard ratio, *CI* confidence interval, *ICD-10* International Classification of Disease, 10th Revision

^a^Significant differences in the adjusted HRs between undiagnosed hypertension and normal blood pressure status determined using the Bland-Altman method

^b^Significant differences in the adjusted HRs between diagnosed hypertension and normal blood pressure status determined using the Bland-Altman method

Compared with education officers, police officers did not show significant differences in the risk of developing CVD following stratifications by sex, BMI, and diabetes status (Tables S2–S4).

## Discussion

This study aimed to investigate the association between police officers and the risk of developing CVD while accounting for socioeconomic and demographic variations and compare this risk with that of education officers. To the best of our knowledge, this is the first population-based study using a matched cohort of police and education officers and nationwide data. The matching process ensured that age, sex, and the calendar year of job enrollment were accounted for, considering their relevance to the development of CVD. The analysis was also adjusted for covariates known to significantly influence CVD risks. The main findings revealed that police officers were at a 1.2-times significantly higher risks of developing CVD compared with education officers, as well as acute myocardial infarction and ischemic stroke. Moreover, there was a significant difference found in the risk of developing CVD among police officers aged ≥45 years old compared to those aged 20–44 years old, as well as between police officers with undiagnosed hypertension and those without hypertension.

Police officers play an important role for maintaining societal security, yet their work environment is pressurized with various stressors that endanger their physical and mental health, particularly in relation to CVD. These stressors include a variety of factors, including encountering death, suffering, poverty, physical threats, and irregular work schedules. Night shifts, essential for their duties, disrupts police officers’ circadian rhythms, meal schedules, and sleeping patterns [8], leading to increased risks of CVD and CVD-related mortality [9–11]. Exposure to noise and particulate matter [12–14], especially for traffic police, also contributed to CVD risks [15–17]. Moreover, emotional stressors, including mental and psychosocial stressors, independently increases the risk of developing CVD [18–22], as demonstrated by a 2022 meta-analysis across 32 countries, which showed increased risks of ischemic stroke and intracerebral hemorrhage in association with emotional stress [22]. Emotional stress is also linked with the onset of diabetes and obesity, thus increasing the susceptibility to CVD [23, 24]. Traumatic events such as accidents and firearm incidents contribute to mental health issues among police officers [25, 26], with a high prevalence of posttraumatic stress disorder found [27]. Furthermore, the climate (temperature and humidity) affects blood pressure and heart rate fluctuations, potentially elevating CVD risks of police officers [28].

Our findings align with previous studies that showed a high prevalence of CVD and CVD-related mortality in police officers [2, 4]. In particular, our study highlighted its association with age and hypertension status. We found a higher risk of developing CVD, acute myocardial infarction, and ischemic stroke among police officers aged 45 and older, with notable differences in risk across age groups. Aging individuals experience cardiac functional changes, including diastolic and systolic dysfunction, electrical abnormalities, and arrhythmias, which predispose older individuals to cardiovascular complications [29]. Age-related oxidative stress, inflammation, and mitochondrial dysfunction also play key roles in cardiac alterations, ultimately leading to impaired cardiac function and CVD development [29]. Of the 104,134 police officers included in the present study, 19,269 officers (18.5%) had hypertension, with a concerning 57.0% (10,991 officers) being undiagnosed with hypertension. This observation is notably higher than the national statistics, which indicate that 40.3% of the adult population aged between 20–65 years old has undiagnosed hypertension [30]. In the present study, police officers with undiagnosed hypertension showed a significantly elevated risks to developing CVD and acute myocardial infarction compared to those without hypertension. Previous study has demonstrated higher prevalence of high blood pressure among police officers (25.7%) compared to individuals in other professional specialties (17.6%), such as education officers, engineers, scientists, health professionals, and artists [31]. Moreover, police officers are less likely to be aware of their elevated blood pressure status and to take medicines to control the condition [32]. Taken together, implementing screening efforts for aging (e.g., individuals in their mid-40s and beyond [1, 33]) and hypertension status may be essential to mitigate CVD risks for police officers.

This study had several limitations. First, the years of job duration for both police and education officers were not considered, which could potentially impact the results. However, a study that used fixed cohorts identified through 3-year consecutive health insurance registration data showed that the standardized incidence ratios of all CVD for police officers were 1.71 (95% CI, 1.66–1.76) when compared with all public officers and 2.10 (95% CI, 2.04–2.17) when compared with general and education officers [4]. Second, in the present study, the prevalence of CVD was higher than the general Korean population [34]; thus, caution is required when interpreting these findings. While Korean National Health Insurance Service claims acute myocardial infarction prevalence rate of 0.3% and the Korea National Health and Nutrition Examination Survey reported 0.7%, this study found a higher incidence of 1.2% among education officers, representing the general Korean population. These discrepancies in prevalence are likely due to the differences in characteristics of the survey data and our matched cohort data. This study was a matched cohort study, with approximately 85% of the population being men. According to the Korean National Health Insurance Service data, men in Korea have 2.4-folds higher incidence of developing acute myocardial infarction [34]. Thus, the higher prevalence of CVD in education officers may be due to the predominance of men in our cohort. Finally, the study did not include information on potential confounders such as particulate matter and noise exposure, nor did it sufficiently report lifestyle habits like physical activity, from the Korean National Health Insurance Service. Future cohort studies specifically designed for investigating this association among police officers are required, using specialized health examination designed for the purpose to understand these effects [35].

## Conclusions

The findings of our study highlight a significant increase in the risk of developing CVD among police officers, particularly among those aged 45 years and older and those with uncontrolled blood pressure compared to their education officer counterparts. Therefore, it is crucial to enhance medical protection measures for these occupational groups to mitigate their susceptibility to CVD.

### Supplementary Information

Additional file 1: Table S1. Distribution of blood pressure and fasting blood glucose in police officers and education officers, stratified by diagnosis status. Table S2. Risk of cerebro-cardiovascular diseases stratified by sex. Table S3. Risk of cerebro-cardiovascular diseases stratified by body mass index. Table S4. Risk of cerebro-cardiovascular diseases stratified by diabetes status

## Data Availability

The present study acquired data from the Korean National Health Insurance Service in the Republic of Korea. The analytic code will be made available from the corresponding authors on request.

## Abbreviations

BMI: Body mass index
CI: Confidence interval
CVD: Cerebro-cardiovascular diseases
HR: Hazard ratio
ICD-10: International Classification of Disease, 10th Revision
