# Supplementary material for: Increased risk of developing cerebro-cardiovascular diseases in police officers: a nationwide retrospective cohort study
Source: Clin Hypertens. 2024 Jul 1;30:18. doi: 10.1186/s40885-024-00277-6 (PMC11215820; doi:10.1186/s40885-024-00277-6)
Supplement: Supplementary file 1 — Additional file 1: Table S1. Distribution of blood pressure and fasting blood glucose in police officers and education officers, stratified by diagnosis status. Table S2. Risk of cerebro-cardiovascular diseases stratified by sex. Table S3. Risk of cerebro-cardiovascular diseases stratified by body mass index. Table S4. Risk of cerebro-cardiovascular diseases stratified by diabetes status [file 40885_2024_277_MOESM1_ESM.docx]

**Table S1.** **Distribution of blood pressure and fasting blood glucose in police officers and education officers, stratified by diagnosis status**

| Biomarkers | Police officers (n=104,134) | | Education officers (n=104,134) | | p |
| --- | --- | --- | --- | --- | --- |
| Systolic blood pressure (mmHg), mean (SD) |  |  |  |  |  |
| Diagnosed hypertension | 129.4 | (14.2) | 129.6 | (15.1) | 0.197 |
| Undiagnosed hypertension | 142.2 | (11.9) | 141.9 | (13.8) | 0.071 |
| No hypertension | 118.8 | (10.7) | 116.9 | (11.2) | <0.001 |
| Diastolic blood pressure (mmHg), mean (SD) |  |  |  |  |  |
| Diagnosed hypertension | 81.7 | (10.0) | 82.2 | (10.6) | <0.001 |
| Undiagnosed hypertension | 91.8 | (8.1) | 92.0 | (8.1) | <0.001 |
| No hypertension | 74.2 | (7.7) | 72.9 | (8.1) | <0.001 |
| Fasting blood glucose (mg/dL), mean (SD) |  |  |  |  |  |
| Diagnosed diabetes | 78.7 | (9.6) | 77.8 | (10.2) | <0.001 |
| Undiagnosed diabetes | 81.2 | (10.1) | 81.6 | (11.4) | 0.291 |
| No diabetes | 76.1 | (9.4) | 74.9 | (9.9) | <0.001 |

*Abbreviations:* SD, standard deviation.

*Footnotes:* p-values were obtained using independent t-tests (for continuous, normally distributed variables). Hypertension status was categorized as diagnosed hypertension, undiagnosed hypertension, and no hypertension. Diagnosed hypertension is defined as having at least one relevant ICD-10 codes for hypertension (I10–I16) at primary and/or secondary positions in the past three years prior to cohort entry and/or systolic blood pressure exceeding 140 mmHg or diastolic blood pressure exceeding 90 mmHg in the year of cohort entry. Undiagnosed hypertension is defined as having systolic blood pressure exceeding 140 mmHg or diastolic blood pressure exceeding 90 mmHg without a prior hypertension diagnosis. Individuals who did not meet the criteria for either diagnosed or undiagnosed hypertension were classified as no hypertension.

Diabetes status was categorized as diagnosed diabetes, undiagnosed diabetes, and no diabetes. Diagnosed diabetes was defined as having at least one relevant ICD-10 code for diabetes (I20–I25, I48, I50, I60–I69) at primary and/or secondary positions in the past three years prior to cohort entry and/or fasting blood sugar levels ≥126 mg/dL in the year of cohort entry. Undiagnosed diabetes is defined as having fasting blood sugar levels ≥126 mg/dL without a prior hypertension diabetes. Individuals who did not meet the criteria for either diagnosed or undiagnosed diabetes were classified as no diabetes.

**Table S2. Risk of cerebro-cardiovascular diseases stratified by sex**

| Disease | Men | | Women | | *p inter^†^* |
| --- | --- | --- | --- | --- | --- |
|  | **Hazard ratio (95% CI)** | | **Hazard ratio (95% CI)** | |  |
| Cerebro-cardiovascular diseases | 1.16 | (0.10–1.22) | 1.25 | (1.02–1.55) | 0.474 |
| Acute myocardial infarction | 1.16 | (1.06–1.27) | 1.57 | (1.08–2.29) | 0.121 |
| Ischemic stroke | 1.17 | (1.09–1.26) | 1.25 | (0.91–1.71) | 0.695 |
| Hemorrhagic stroke | 1.06 | (0.93–1.21) | 1.02 | (0.68–1.54) | 0.872 |

*Abbreviations:* CI, confidence interval.

*Footnotes:* Adjusted hazard ratios were estimated using multivariable Cox regression models, including adjustments for age, body mass index, health insurance rate, total cholesterol, alcohol consumption, smoking status, hypertension and diabetes statuses. Total cholesterol variables were log-transformed.

^†^ p for interaction: Significant differences in the adjusted hazard ratios between men and women determined using the Altman and Bland method.

**Table S3. Risk of cerebro-cardiovascular diseases stratified by body mass index**

| Disease | Normal (BMI <25 kg/m^2^) | | Obese (BMI ≥25 kg/m^2^) | | *p inter^†^* |
| --- | --- | --- | --- | --- | --- |
|  | **Hazard ratio (95% CI)** | | **Hazard ratio (95% CI)** | |  |
| Cerebro-cardiovascular diseases | 1.12 | (1.04–1.20) | 1.20 | (1.11–1.29) | 0.210 |
| Acute myocardial infarction | 1.10 | (0.98–1.24) | 1.23 | (1.08–1.40) | 0.223 |
| Ischemic stroke | 1.17 | (1.05–1.29) | 1.17 | (1.05–1.29) | 0.983 |
| Hemorrhagic stroke | 0.96 | (0.81–1.13) | 1.23 | (1.01–1.50) | 0.060 |

*Abbreviations:* BMI, body mass index; CI, confidence interval.

*Footnotes:* Adjusted hazard ratios were estimated using multivariable Cox regression models, including adjustments for age, sex, body mass index, health insurance rate, total cholesterol, alcohol consumption, smoking status, hypertension and diabetes statuses. Total cholesterol variables were log-transformed.

^†^ p for interaction: Significant differences in the adjusted hazard ratios determined using the Altman and Bland method.

**Table S4. Risk of cerebro-cardiovascular diseases stratified by diabetes status**

| Disease | No diabetes | | Undiagnosed diabetes | | Diagnosed diabetes | | *p inter^a^* | *p inter^b^* |
| --- | --- | --- | --- | --- | --- | --- | --- | --- |
|  | **Hazard ratio (95% CI)** | | **Hazard ratio (95% CI)** | | **Hazard ratio (95% CI)** | |  |  |
| Cerebro-cardiovascular diseases | 1.16 | (1.10–1.23) | 1.30 | (0.98–1.72) | 1.10 | (0.97–1.26) | 0.462 | 0.490 |
| Acute myocardial infarction | 1.15 | (1.05–1.27) | 1.46 | (0.90–2.37) | 1.16 | (0.92–1.46) | 0.351 | 0.974 |
| Ischemic stroke | 1.17 | (1.07–1.26) | 1.22 | (0.85–1.75) | 1.20 | (1.01–1.42) | 0.815 | 0.758 |
| Hemorrhagic stroke | 1.10 | (0.96–1.26) | 1.19 | (0.60–2.38) | 0.76 | (0.52–1.10) | 0.822 | 0.066 |

*Abbreviations:* CI, confidence interval.

*Footnotes:* Adjusted hazard ratios were estimated using multivariable Cox regression models, including adjustments for age, sex, body mass index, health insurance rate, total cholesterol, alcohol consumption, smoking status, and hypertension status. Total cholesterol variables were log-transformed.

*p inter^a^*: Significant differences in the adjusted hazard ratios between undiagnosed diabetes and normal glycemic status determined using the Altman and Bland method.

*p inter^b^*: Significant differences in the adjusted hazard ratios between diagnosed diabetes and normal glycemic status determined using the Altman and Bland method.
